# Atlanta Academy of Medicine

**Published:** 1872-07

**Authors:** J. T. Johnson

**Affiliations:** Reporter


### ATLANTA ACADEMY OF MEDICINE.

J. T. JOHNSON, M.D., REPORTER.

[Extract from Minutes, May 6th, 1872—Dr. J. P. LOGAN in the Chair.]

Dr. H. K. Smith, Surgeon United States Army, gave the particulars of a case of hemiopia, in one of the soldiers at the barracks, in which the one-sixtieth part of a grain of strychnia, three times a day, had effected a cure in three days. He further added, that Dr. Chisolm, of Baltimore, and others, have recently advocated the use of hypodermic injections of strychnia in this affection, reporting some cases wherein the effect was very marked. They have used it in doses considerably larger than there required for the cure of this patient. Dr. Smith also made interesting remarks as to the character of the disease, and its frequency on the Western plains, among sailors, etc.; also the means, supposed by these persons, efficacious in its cure.

Dr. C. A Simpson, at call of the President, gave the history of the disease, and its treatment, as occurring under his observation. Had seen the treatment by strychnia, hypodermically, very successful; doses ranged from one-fiftieth to one-twentieth of a grain, and even larger. This agent seems to act with special power on the optic nerve. The condition of the patients was usually such as to call for general tonic and invigorating treatment. Is himself now treating a patient for amblyopia, by strychnia; also by iodide of potassium. The choroid is principally affected; the optic nerve appears only a little pale. There is but little, if any, improvement yet.

Dr. John Stainback Wilson desired to know if there was any evidence as to the permanency of the good effects thus exerted on the eye; himself thought not, reasoning from the analagous effects of strychnia in paralysis. Would suggest cold water as a permanent tonic, and, at the same time, an immediate invigorator.

Dr. Simpson repliied that he had not heard anything disproving its permanency. Had' opportunity for observing a number of cases for several weeks; there was no relapse in these.

Dr. J. T. Johnson reported a case of chloroform poisoning he had recently attended. The patient, a white woman, tired of living, had swallowed nearly six drachms, as ascertained by careful investigation. Arrived half hour, or an hour, after she had taken it. Her condition: pulse sixty-eight, full; respiration thirty-six, and imperfect; pupils dilated. Would not have been objectionable, if given by inhalation for a severe and painful operation. We would then withhold it; but here the whole quantity was in the blood. Knowing no special antidote to neutralize it, resolved on endeavoring to sustain the powers of the system until the poison could be eliminated by the different organs of the body. Symptoms of sinking coming on, administered an ounce of brandy by enema, and nearly three-fourths of a grain of morphia subcutaneously, as the most direct stimulant. The effect was immediate and marked; the patient rallied, but depressive sinking again threatened in an hour or two, to be as promptly relieved by a repetition of the same treatment. Six hours after swallowing it, she gave the first sign of consciousness. Gastritis, rather slight, supervened, but moderated in three or four days; for this, little treatment was prescribed. Patient objected to further use of hypodermic medication. Ice was given. Oxalate of cerium, in two and a half grain doses, repeated five times in about three hours, and continued afterward, seemed to control the retching and vomiting—at least, she suffered no more after its use.

Dr. H. B. Lee states that he also saw the case—was called in the absence of Dr. Johnson; found patient's condition as just described. Would have used ammonia by inhalation and per orem, in addition to the other remedies, as a powerful and prompt stimulant.

Dr. Wilson thinks this an interesting case, in showing that we may give full doses of chloroform with safety. Has himself taken as much as one drachm at a dose. Recently saw a case where a man was supposed to have taken four ounces of chloroform; he appeared wild and excited, as if having been on a spree. A woman was going after another supply for him; she exhibited a four-ounce bottle, saying she thought the man had emptied it.

Dr. Ray asked the opinion of the President as to the anti-hemorrhagic properties of ergot.

President Logan replied, that as the general result of his observation, he had great confidence in it—rather referring this action to its contractile powers, than to direct styptic effect.

Dr. Boring, at call of the President, made a report of the use of ergot in a case just concluded. At daylight, saw a woman, three months pregnant, who was about suffering abortion. Hemorrhage had been profuse through the night. Gave ergot; pains increased, and hemorrhage ceased. In half an hour, another dose; this had a sedative effect, pains becoming less violent. Another half hour, another dose; effect yet more decidedly sedative, the pains ceasing entirely. After she slept two or three hours, passed his finger, and drew away what he first thought a polypus; it proved to be what is called a "mole," a kind of false conception. There was no sign of attachment by cord. It was as large as a hen's egg.

Dr. Smith stated that he had a precisely similar case last winter. The pains were hard, the flowing profuse. Gave a drachm of tincture of ergot; this arrested both the hemorrhage and pains. The patient went to sleep, and so did he. Two nights afterward, he was again sent for, and found that a blighted ovum had been expelled. It was similar in appearance to a piece of liver. After the patient got well, was told that she had, in the first month of pregnancy, taken extract of cotton root. Thinks the ovum was then killed, but went on with a spurious development till its expulsion.

Dr. Ray remarked, that he had never seen any results from ergot. Had ceased to administer it; had no faith in it; would as readily rely on a dose of ice-water.

Dr. W. N. Judson said his experience coincided with Dr. Ray's. Used it a number of times, but had never known it to produce the least effects. Perhaps, though, he was unfortunate in obtaining a poor article of the medicine.

Dr. Raushenberg had often seen violent and almost incessant contractions of the uterus produced by the ordinary preparations of ergot. Alluded to the use of ergotin in varicose veins. Thought the effect produced by its influence on the muscular tissue; has not seen such effect claimed as produced on larger vessels. Is convinced that ergot exercises a controlling influence on capillary hemorrhage. Has several times used it with good effect in hæmoptysis. Thinks it would be a good remedy in meningitis, and advanced theories and reasons for his faith in its adaptability to the treatment of the same.

Dr. W. T. Grant (visiting the Academy) was requested to give his views on the questions discussed.

He said that, some time since, while suffering from a headache so agonizing as to make death as welcome as its continuance, he had taken two table-spoonsful of chloroform in an hour or little more. No evil effect followed. As to ergot, had never derived any benefit from it in obstetrics. Thought it would prove useful in meningitis, but had no opportunity for testing it. Had recently seen a number of peculiar diseases of the eye, all occurring among the hands employed in building railroads; overseers told him they had seen hundreds of such cases. It was an amaurosis, affecting the patient only at night; when the sun went down, the patient became blind, and so remained till daylight. No treatment was instituted, as all recovered in a few days without it.

